# Supplementary material for: Prevalence and associated factors of treatment failure among children on ART in Ethiopia: A systematic review and meta-analysis
Source: PLoS One. 2022 Apr 14;17(4):e0261611. doi: 10.1371/journal.pone.0261611 (PMC9009617; doi:10.1371/journal.pone.0261611)
Supplement: Attachment — Submitted filename: Response to Reviewers.docx [file pone.0261611.s003.docx]

**Dear Editor of PLOS ONE editorial office**

This is a point-by-point response letter that accompanies the responses of authors to reviewers’ comments concerning to our manuscript. I am pleased to resubmit the revised version of our paper entitled “**Prevalence and associated factors of treatment failure among children on ART in Ethiopia: a systematic review and meta-analysis”** for a review as original research in **PLOS ONE**. We have taken advantage of the academic editorial vital comments and views to improve our manuscript as per the journal requirements. In the document, we have amended “no funding was obtained for this review” as “the authors received no specific funding for this work” and “data from this study will be available up on request” as “data from this study will be available from the supporting information file”.

It is well-known that the manuscript has been reviewed by peer reviewers and sent back to authors for first revision and resubmission. We would like to take this opportunity to acknowledge the reviewers for their valuable and constructive comments. The reviewers’ comments were insightful and enabled us to improve the quality of our manuscript significantly. We are so happy and thankful to work with you. The point-by-point responses for each of the reviewers’ comments are addressed in the following pages using a point by point format. The authors’ responses are also shown by the track changes in the revised version of our manuscript. **Our responses are written in blue font color.**

We look forward to hearing from you at your earliest convenience.

Kind regards

Belete Gelaw

(On the behalf of other authors)

**Reviewer #1**

**Comments to the Author**

The authors of this systematic review and meta-analysis have presented useful data to determine the pooled national burden of childhood HIV/AIDS treatment failure as well as its associated factors in Ethiopia. Conversely, there are specific comments that the reviewer would like the authors to address so as to further improvement of the manuscript.

Authors’ response: we are very glad to the reviewer’s appreciation of our efforts; and we have just given our respective responses to each of the specific reviewer comments as detailed below.

1. **Is the manuscript technically sound, and do the data support the conclusions?**

Thank you very much for your insightful comment. Yes. We have a conclusion which is supported by the data analysed and presented in this study (page 16, line 327-336).

2. **Has the statistical analysis been performed appropriately and rigorously?**

We acknowledge the reviewer’s concern regarding to statistical analysis for this study. Yes. We have carried out all the necessary statistical analysis properly and presented our finding accordingly in the main documents of this study.

3. **Have the authors made all data underlying the findings in their manuscript fully available?**

Yes. **Comments have been taken and we have made the necessary correction accordingly (page 18, line 357-358). For detail please see the supporting information file.**

4. **Is the manuscript presented in an intelligible fashion and written in standard English?**

Great thanks. We have carefully reviewed the manuscript based on your suggestion and some typographic error has been also updated (page 7, line 153).

5. **It will be worthwhile to do a subgroup/sensitivity analysis of cohort and cross-sectional analysis. As, in you may exclude cross-sectional studies and check for the estimates in cohort studies. Repeat it for cross-sectional and compare whether there is a difference. You may also look at Urban/rural population. Any variable for meta-regression?**

Absolutely! We have taken the given comment and sensitivity analysis was already done accordingly. However, the result indicated that there is no single study unduly influenced the overall estimates of treatment failure and there is no significant variability for meta-regression. We have done subgroup analysis based on study design, and giving a prevalence of 12.14(95% CI: 7.83, 16.44) in cohort which is nearly the same in cross-sectional study 13.43 (95% CI: 10.76, 16.11) (page 12, line 226-237).

NB. Generally, we have tried to revise our manuscript according to the requirement of PLOS ONE template.
